# Upregulation of human β-defensin-3 and cathelicidin LL-37 in Kaposi’s sarcoma

**DOI:** 10.12688/f1000research.1-38.v2

**Published:** 2012-12-14

**Authors:** Hanan Fathy, Maha M Amin, Abdel-Hady El-Gilany

**Affiliations:** 1 Faculty of Medicine, Mansoura University, Mansoura, Egypt

## Abstract

**Background:** Kaposi’s sarcoma (KS) is a rare neoplasm of lymphatic endothelial cells. Human herpes virus 8 (HHV-8) is considered to be a necessary, but not sufficient causal agent of KS and additional cofactors remain unknown. In this study we evaluated the expression of human β defensin (HBD)-3 and LL-37 in cutaneous lesions of KS in comparison to the healthy skin of normal subjects.

**Methods: **We performed a quantitative immunohistochemical study of HBD-3 and LL-37 on skin lesions from 18 patients having KS, and on healthy skin from 12 normal controls.

**Results:** HBD-3 and LL-37 were significantly upregulated in epidermal and dermal specimens of all KS patients in comparison to normal skin of healthy controls. The immunostaining score of dermal HBD-3 was significantly higher in nodular lesions (9.6 ± 2.4) versus plaque lesions (4.1 ± 2.2), P = 0.001. Also the immunostaining score of dermal LL-37 was significantly higher in nodular lesions versus plaque lesions (P = 0.001).

**Conclusions:** We have demonstrated for the first time that HBD-3 and LL-37 are significantly upregulated in lesional skin of KS in comparison to the skin of healthy controls. The obtained data suggest a possible involvement of these antimicrobial peptides in the pathogenesis of KS. However, the biological significance of HBD-3 and LL-37 in KS lesions needs further research.

## Background

Kaposi’s sarcoma (KS) is a rare disease of lymphatic endothelial cells frequently evident as multiple vascular cutaneous and mucosal nodules. Lymph node and visceral manifestation is seen in cases of strong immunosuppression or aggressive disease ^1^. The four clinico-epidemiological forms of KS are: a classic form typically affecting elderly men of the Mediterranean, the endemic presence in Southern Africa, the epidemic form in patients infected with human immunodeficiency virus (HIV) and the iatrogenic KS complicating iatrogenic immunosuppression ^2^.

KS is strongly associated with human herpes virus 8 (HHV-8), which is implicated in the pathogenesis of all forms of KS ^3^. HHV-8 is present in the vast majority (> 90%) of spindle cells and in the neoangiogenic vessels ^4– 6^. It is considered to be a necessary, but not sufficient causal agent of KS. Besides immunosuppression and AIDS, additional cofactors remain unknown ^7^.

Ensoli *et al.* ^8^ speculated that early stage KS is a reactive inflammatory angiogenic process that may be triggered or enhanced by infection with HHV-8 with many lymphocytes and monocytes infiltrating the lesion. These cells produce inflammatory cytokines including interferon-y (IFN-γ), tumor necrosis factor-α (TNF-α), interleukin-1β (IL-1β), IL-2, IL-6 and others ^9^. The inflammatory cytokines induce the recruitment of circulating cells into tissues, induce the production of angiogenic factors that mediate angiogenesis and edema and activate endothelial cells to acquire the phenotype of KS spindle cells ^10^.

Antimicrobial peptides (AMPs) are an evolutionarily conserved component of the innate immune system that defend against invading bacteria, viruses and fungi through membrane or metabolic disruption ^11^. This diverse group of peptides is separated into several classes. Defensins and cathelicidin (also known as LL-37) are considered the two main groups of AMPs in human skin ^12^. It was confirmed that human β defensin (HBD)-3 and LL-37 demonstrated the ability to inhibit viral infection, affecting both enveloped RNA and DNA viruses and non-enveloped viruses ^13– 21^. Moreover, beside their role in protecting the host from the invasion of pathogens, HBD-3 and LL-37 have immunomodulatory properties in inflammatory skin diseases as psoriasis ^22, 23^ and rosacea ^24^.

Based on the antiviral and immunomodulatory effect of HBD-3 and LL-37, as well as the angiogenic role of LL-37 ^25^, this study was carried out to assess the expression of the two main antimicrobial peptides (HBD-3 and LL-37) in skin lesions of KS in comparison to normal skin from healthy subjects.

## Materials and methods

### Patients and controls

This is a case control study including a convenient sample of 18 Egyptian patients with KS recruited sequentially from the outpatient clinic of the Dermatology unit of Mansoura University Hospital from 2006 to 2011. The diagnosis was based on classic clinical, histopathological features of KS and immunohistochemical staining using the endothelial marker CD34. The controls were 12 healthy subjects that underwent surgical removal of benign lesions.

### Clinical evaluation and skin samples

A thorough general and skin examination of patients was done, focusing on morphology, localization and number of skin lesions, as well as oral mucosa and lymph nodes examination. A blood cell count, blood chemistry, ELISA test for HIV serology, chest X-ray and abdominal ultrasound were conducted. As classic KS rarely affects other organs ^26^, gastrointestinal tract endoscopies were done only to patients with widespread skin lesions (four patients) to evaluate the presence of gut lesions. Inclusion criteria were newly diagnosed patients without previous treatment of KS. Only patients with fully developed lesions such as plaques and nodules were included in this study. Patch (macular) stage lesions were not included. Staging of KS was assigned using the classification proposed by Schwartz *et al.* ^27^ and modified by Schwartz *et al.* ^28^ as follows:

- **Stage I:** Localized nodular KS, with ≤ 15 cutaneous lesions or involvement restricted to one bilateral anatomic site, and few, if any gut nodules.
- **Stage II:** Includes both exophytic destructive KS and locally infiltrative cutaneous lesions and locally aggressive KS or nodular KS, or > 15 cutaneous lesions or involvement of more than one bilateral anatomic site, and few or many gut nodules.
- **Stage III:** Widespread lymph node involvement, with or without cutaneous KS, but with limited if any visceral involvement.
- **Stage IV:** Widespread KS, usually progressing from stage II or III, with involvement of multiple visceral organs with or without cutaneous KS.

A lesional skin biopsy (intact lesion without evidence of secondary infection) was obtained from each patient. Biopsy from 12 normal subjects served as controls. They were obtained from normal skin beside benign neoplasms such as melanocytic naevus, infundibular cyst and lipoma of age, sex and localization-related subjects. Informed consent was taken from all participants. The study protocol was approved by the ethical committee of the College of Medicine of Mansoura University.

### Immunohistochemistry

All specimens were fixed in formalin 10% and sections from paraffin blocks (3–4 um) were cut on glass slides for routine hematoxylin and eosin stains as well as immunohistochemical staining using an bindirect avidin-biotin-peroxidase method. Endogenous peroxidase activity was blocked with 0.6% H _2_O _2_. After blocking, sections were incubated at room temperature for 60 minutes with antibodies to HBD-3 using rabbit polyclonal antibody (Catalog number D2444; Sigma Aldrich, St. Louis, USA) at a dilution of 1:2 and with human LL-37 monoclonal antibody (Catalog number HM2070; Hycult Biotech, Frontstraat 2a, 5405 PB Uden Netherland) at a dilution of 1:500. Diaminobenzidine (DAB) reaction was used for visualization, followed by a hematoxylin counterstain. Negative controls for all studies were obtained by omission of the primary antibodies of an adjacent section to assess the degree of non-specific staining. All slides were examined by Olympus light microscope.

### Immunohistochemical analysis of HBD-3 and LL-37

Immunostaining results of HBD-3 and LL-37 were evaluated in four layers of the epidermis and the proposed score was modified from Meyer-Hoffer *et al.* ^20^ as follows: 0, none; 1, stratum corneum only; 2, stratum corneum and stratum granulosum; 3, stratum corneum, stratum granulosum and stratum spinosum; 4, whole epidermis. Next an intensity score was assigned, which represented the average intensity of positive epidermal cells as follows: 0, none; 1, weak; 2, moderate; 3, intense staining. Both scores were then added to obtain a total epidermal score which ranged from 0 to 7. Skin samples of psoriasis that are known to exhibit high expression of HBD-3 and LL-37 in the epidermis were used as positive controls. Immunostaining results of dermal lesions of KS were scored as previously described ^29^. Immunoreactivity of HBD-3 and LL-37 were evaluated in the two basic component of this disease (spindle-shaped cells and endothelial cells of newly formed vessels). The percentage of positive cells was graded from 0 to 4 as follows: 0, zero to 10%; 1, 11 to 33%; 2, 33 to 66%; 3, 67 to 90%; and 4, 91 to 100%. Specimens were considered immunopositive when more than 10% of cells showed clear evidence of immunostaining. The intensity of immunostaining was rated as follows: 0, none; 1, weak; 2, moderate; and 3, intense. Because KS lesions frequently showed significant intra-specimen heterogeneity, a score was calculated in which the percentage positive rating was multiplied by the intensity rating. Each component of the lesion was scored independently and the results were added up. The score was calculated on 6 to 10 representative high–power fields after examination of the totality of lesion present in one section for each case. Sweat glands served as internal positive controls for LL-37. Inflammatory cells (monocytes and macrophages) served as internal positive controls for both studied AMPs. However, specimens from healthy subjects served as negative controls. Slides were scored by two independent and trained researchers (the authors).

### Statistical analysis

Data were analyzed using SPSS version 16. Qualitative variables were presented as numbers and percentages. Quantitative variables were presented as mean ± SD (median) and a Mann-Whitney test was used for group comparison. Spearman’s correlation coefficient was used to calculate correlation between variables in patients with KS. P ≤ 0.05 was considered statistically significant.

## Results

The mean age of the patients (fourteen males and four females) was 63.9 ± 7.4 years (ranged from 55 to 82 years). The age and sex-matched control group were eight males and four females with a mean age of 63.2 ± 3.7 years (ranged from 45 to 67 years). All investigated patients had classic KS without clinical evidence of immunodeficiency and no history of immunosuppressive drug intake. They had normal total and differential leukocytic count, normal blood chemistry and negative HIV serology. Fourteen KS patients were classified as stage I (≤ 15 cutaneous lesions) and 4 patients as stage II (> 15 cutaneous lesions) ( Table 1). No patients had mucosal lesions, lymphadenopathy or clinical or radiological features suggesting visceral involvement and none had gut nodules as determined by endoscopy (which was only done to stage II KS patients).

**Table 1.** Demographic, clinical features of studied patients and mean scores of immunostain of HBD-3 and LL-37.

Feature	Mean ± SD/Number (%)
Age (year)	63.9 ± 8.04
Sex (No & %) Males Females	14 (77.8%) 4 (22.2%)
Duration of lesions (months) Minimum – Maximum	7.1 ± 4.5 1 – 14
*Site of KS lesions Feet and legs Upper limbs Trunk Face	18 (100%) 4 (22.2%) 2 (11.1%) 2 (11.1%)
Type of skin lesions biopsied Plaques Nodules	8 (44.4%) 10 (55.6%)
Tumor stage Stage I Stage II	14 (77.8%) 4 (22.2%)
Epidermal HBD-3	4.4 ± 1.6
Dermal HBD-3	7.2 ± 3.6
Epidermal LL-37	4.5 ± 1.7
Dermal LL-37	6.7 ± 3.1

Kaposi’s sarcoma (KS), human b defensin (HBD)-3, cathelicidin (LL-37).

* Some patients had many anatomic sites affected.

**Figure 1.**
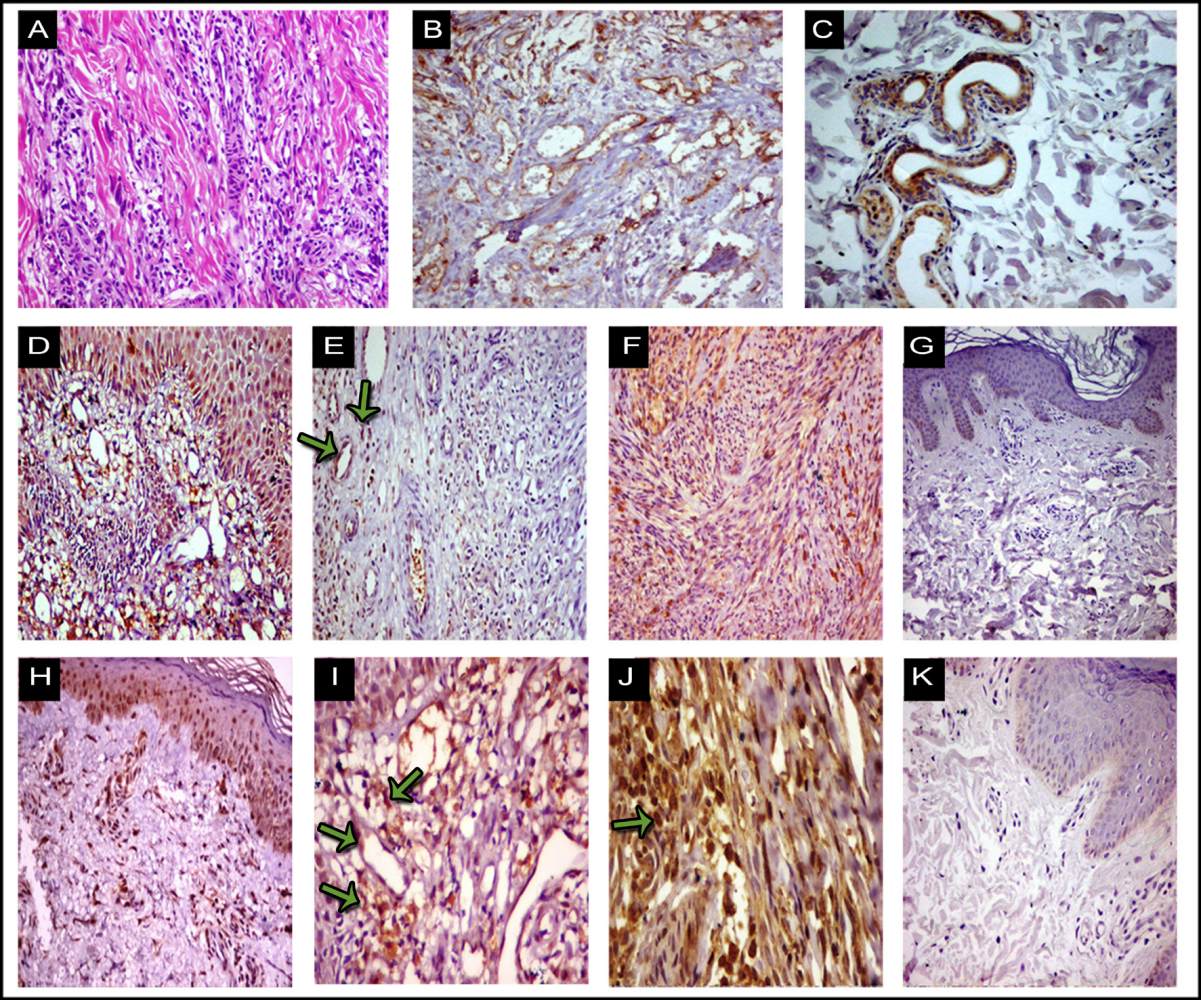
Representative images of Kaposi’s sarcoma (KS). Lesions with hematoxylin and eosin stain ( **A**), CD34 ( **B**), human β defensin (HBD)-3 ( **D-G**) and cathelicidin LL-37 ( **C**, **H-K**). Immunohistochemistry for CD34 ( **B**) is strongly positive in KS lesions. HBD-3 and cathelicidin LL-37 immunoreactivity is less intense in plaque ( **E**, **I**; respectively) than in nodular lesions ( **F**, **J**; respectively). Positive immunohistochemical staining of HBD-3 and LL-37 in endothelial cells and spindle shaped cells are indicated by arrows. Positive epidermal staining for HBD-3 ( **D**) and cathelicidin LL-37 ( **H**) can be appreciated. Positive internal control for cathelicidin LL-37 in sweat glands ( **C**) and inflammatory cells ( **E**, **I**) for HBD-3 and cathelicidin LL-37; respectively are seen. Healthy skin as negative control for HBD-3 and cathelicidin LL-37 are seen in ( **G**, **K**). Original magnification: ( **A**, **B**, **C**, **D**, **E**, **F**, **H**, **K**) X 200; ( **G**) X 100; ( **I**, **J**) X 400.

Immunohistochemical staining of HBD-3 and LL-37 were generally cytoplasmic, and membranous staining was also seen. There was significant upregulation of HBD-3 and LL-37 in both epidermal and dermal specimens of all studied patients in comparison to normal skin of healthy controls. The expressions of HBD-3 and LL-37 were seen in the epidermis as well as in the dermis (neoformed vessels, spindle cells and inflammatory cells) ( Figure 1D, 1H) with different scores of immunoreactivity in KS lesions ( Figure 1). Also positive immunohistochemical staining for LL-37 was seen in the sweat glands of KS lesions ( Figure 1C). HBD-3 and LL-37 were absent in the epidermis and dermis of control normal skin ( Figure 1G, 1K) Only three sections of control normal skin showed weak staining within the stratum corneum for HBD-3 and only two sections for LL-37 within stratum granulosum.

The immunostaining scores of HBD-3 and LL-37 are detailed in Table 1 and Table 2. We found that in nodular lesions, the mean scores of epidermal HBD-3 (5.4 ± 1.4) and LL-37 (5.6 ± 1.1) were significantly higher in comparison to mean scores of epidermal HBD-3 (3.3 ± 0.9) and LL-37 (3.1 ± 1.2) in plaque lesions ( Table 2). The mean scores of dermal HBD-3 (9.6 ± 2.4) and LL-37 (8.9 ± 1.7) in nodular lesions were significantly higher compared to mean scores of HBD-3 (4.1 ± 2.2) and LL-37 (4.1 ± 2.2) in plaque lesions. Furthermore, the mean scores of epidermal and dermal HBD-3 and LL-37 immunoreactivity were significantly higher in KS patients with stage II disease in comparison to patients with stage I ( Table 2). There was a strong positive significant correlation between immunostaining scores of epidermal and dermal HBD-3 in KS lesions (r = 0.95 and P ≤ 0.001). Also a strong positive correlation between immunostaining scores of epidermal and dermal LL-37 in KS lesions was found (r = 0.7 and P = 0.001) ( Table 3).

**Table 2.** Immunostain of HBD-3 and LL-37 regarding type of skin lesions and tumor stage.

	Epidermal HBD-3 Mean ± SD (Median)	Dermal HBD-3 Mean ± SD (Median)	Epidermal LL-37 Mean ± SD (Median)	Dermal LL-37 Mean ± SD (Median)
Type of skin lesions Plaques (8) Nodules (10) Significant test	3.3 ± 0.9 (3.5) 5.4 ± 1.4 (6.0) Z = 2.7, P = 0.007	4.1 ± 2.2 (5.0) 9.6 ± 2.4 (10.0) Z = 3.3, P = 0.001	3.1 ± 1.2 (3.0) 5.6 ± 1.1 (6.0) Z = 3.1, P = 0.002	4.1 ± 2.2 (5.0) 8.9 ± 1.7 (8.5) Z = 3.4, P = 0.001
Tumor stage Stage I (14) Stage II (4) Significant test	3.8 ± 1.3 (4.0) 6.5 ± 0.6 (7.0) Z = 3.0, P = 0.002	5.9 ± 3 (6.0) 11.5 ± 0.5 (11.5) Z = 2.7, P = 0.007	3.9 ± 1.4 (4.0) 6.5 ± 0.6 (7.0) Z = 3.0, P = 0.003	6 ± 2.9 (6.0) 9.5 ± 1.7 (9.5) Z = 2.1, P = 0.04

**Table 3.** Correlation between immunostaining scores of epidermal and dermal HBD-3 and LL-37.

	Epidermal HBD-3		Epidermal LL-37	
	r	P	r	P
Dermal HBD-3	0.95	≤ 0.001		
Dermal LL-37			0.7	= 0.001

Demographic, clinical and immunohistochemical scoring of HBD-3 and LL-37 of studied population Age in years Sex: M=male, F=female Duration of lesion in months

## Discussion

Recently many reports about the antiviral effect of defensins and cathelicidin have been published. The antiviral activity of HBD-3 has been reported against herpes simplex virus (HSV) ^13^, vaccinia virus ^14^, and HIV ^15^. Similarly LL-37 is found to inhibit replication of vaccinia virus ^16^, kill HSV ^17^, is effective against adenovirus ^18^ and inhibits HIV-1 replication in peripheral blood mononuclear cells ^19^. Furthermore, HBD-3 expression was shown to be upregulated in human papillomavirus induced lesions ^20, 21^.

In our study we investigated for the first time the expression of HBD-3 and LL-37 in KS. They were significantly upregulated in epidermal and dermal (neoangiogenic vessels, spindle cells and inflammatory cells) regions of all studied KS lesions in comparison to normal skin of healthy subjects. These findings suggest a potential role of HBD-3 and LL-37 in the pathogenesis of KS. Furthermore, we found that the expressions of these AMPs were increased with progression of KS lesions from plaque stage to nodules. The stage-related differences that we found in HBD-3 and LL-37 expression add more support to the possible role of these AMPs in the progression of KS.

The induction of HBD-3 and LL-37 in KS may be in response to infection with HHV-8 that is implicated in the pathogenesis of all forms of KS ^3^. HBD-3 and LL-37 might exhibit antiviral activity against HHV-8 as shown for the related HSV ^13, 17^.

In addition to functioning as direct antimicrobial compounds, AMPs can function as chemokines ^30^. It was found that LL-37 increases natural killer cell proliferation by activating the Toll like receptor 9. LL-37 increases proinflammatory cytokines at the dendritic cell level, promoting CD4 ^+^ TH1 cell responses ^31^. LL-37 can synergize with IL-1β to increase the production of cytokines, such as IL-6, IL-8 and IL-10, and chemokines, such as the cc-chemokine ligand 2 ^32^. Similarly to cathelicidin, HBD-3 has chemoattractant properties on different cell types such as T lymphocytes and dendritic cells ^33^. Furthermore, HBD-3 and cathelicidin induce the production of diverse chemokines and cytokines such as monocyte chemotactic protein-1, macrophage inflammatory protein-3, interferon-inducible protein-10, IL-1, IL-6, IL-8, IL-10 and TNF-α mainly in keratinocytes ^34, 35^.

Taken together, it is possible that HBD-3 and LL-37 can function in KS by promoting Th1 cell responses or by increasing the production of cytokines such as IL-1, IL-6, IL-8 and TNF-α. Several cytokines have been shown to support the growth of cultured KS spindle cells: these include IL-1β, IL-6, the soluble IL-6 receptor 2 and TNF-α ^36^.

HHV-8-specific cytotoxic T-lymphocyte and T helper responses are found in KS patients, and CD4 and CD8 ^+^ T cells were present in KS lesions. Also, monocytes-macrophages and dendritic cells were present in lesions ^8^. Furthermore, Sirianni and coworkers ^37^ had shown that NK cell function is important for the control of latent HHV-8 infection and abrogation of this important immune response can lead to a more aggressive KS disease. Moreover, besides LL 37’s function as chemoattractant to T-cells and monocytes, it can stimulate angiogenesis through an increase in endothelial cell proliferation and vessel formation ^25^. AMPs can indirectly sustain angiogenic signals by production of TNF-α and IL-1. These cytokines are powerful inducers of vascular endothelial growth factors ^38^. So the implicated role of HBD-3 and LL-37 in KS may be inhibition of HHV-8 replication, production of several inflammatory cytokines and stimulation of angiogenesis. Ensoli *et al.* ^8^ speculated that KS is a multistep process including not only HHV-8 infection, but also genetic and angiogenic factors, as well as the production of several inflammatory cytokines. Furthermore, Kawsar *et al.* ^39^ reported that HBD-2 is expressed in vascular endothelial cells associated with KS. The authors suggested that HBD-2 may play a role in tumor angiogenesis and metastasis.

It remains unclear whether KS itself is a true malignancy, a reactive proliferation or both ^28^. Whatever the nature of KS, HBD-3 and IL-37 may serve as a growth factor for KS. This is supported by the finding that the expression of these AMPs were increased with progression of KS lesions from plaque stage to nodules in our study. Recent evidence suggests cathelicidin LL-37 to be a putative growth factor for various human cancers ^40– 43^. Similarly, HBD-3 may play an important role in the development and progression of oral cancer. HBD-3 stimulated the expression of tumor-promoting cytokines, including IL-1α, Il-6, Il-8 and TNF-α in macrophages ^44^. These cytokines are also important in the progression of KS ^36^. Further study to elucidate the hypothesis that HBD-3 and LL-37 serve as progression factor for KS is therefore recommended.

It is not known whether upregulation of HBD-3 and LL-37 in KS lesions is signaled indirectly by locally produced proinflammatory cytokines or directly by HHV8 molecules. HBD-3 is upregulated by proinflammatory cytokines, TNF-α, IFN-γ and IL-1β ^22^. However IL-6 is a potent inducer of LL-37 ^45^. Interestingly, these inflammatory cytokines are abundant in KS lesions ^9^. Furthermore, the possibility of induction of these AMPs by HHV-8 protein can be supported by the finding of increased expression of HBD-3 and LL-37 with progression of KS lesions from plaque into nodular stage in our study. Similarly, increased load of HHV-8 has been found with progression of KS lesions ^5^. Further study is recommended to verify this point.

## Conclusions

HBD-3 and LL-37 are for the first time shown to be significantly upregulated in KS skin lesions as compared with skin of healthy controls. The obtained data suggest a possible contribution of HBD-3 and LL-37 in the innate and adaptive immune response target against HHV-8. Another possibility is the potential involvement of these antimicrobial peptides in the pathogenesis of KS. However, the biological significance of HBD-3 and LL-37 in KS lesion needs further research.

## Consent

Written informed consent for publication of clinical details was obtained from the patients.
