# Supplementary material for: Upregulation of human β-defensin-3 and cathelicidin LL-37 in Kaposi’s sarcoma
Source: F1000Res. 2012 Dec 14;1:38. Originally published 2012 Oct 26. [Version 2] doi: 10.12688/f1000research.1-38.v2 (PMC3782342; doi:10.12688/f1000research.1-38.v2)
Supplement: Peer review history

# Peer review: Referee response for version 1

**Published:** 2012-11-19

**Authors:** Barbara Ensoli

**Affiliations:** 1 Director, National AIDS Center, Istituto Superiore di Sanit, Rome, Italy

In this study the authors have evaluated for the first time the expression of human β defensin (HBD)-3 and LL-37 in cutaneous lesions of KS and showed that these antimicrobial peptides (AMPs) are significantly up-regulated in KS skin lesions as compared with skin of healthy controls. The results are of potential interest to the understanding of KS pathogenesis, however there are several points that should be addressed which can improve the work.

1. The authors should explain why they excluded from the immunohistochemical evaluation macular lesions considering that these lesions are the one with the highest degree of inflammatory infiltrate, and given the role of the peptides in inflammatory skin diseases.

2. It is not clear how the scoring for the immunohistochemical analysis was calculated (see Material and Methods section). In particular it seems that a total epidermal score (ranging from 0 to 7) was obtained by the addition of a score representing the extension of the expression detected in the four epidermal layers with an intensity score, which represented the average intensity of positive epidermal cells. Then the percentage of positive cells is calculated, and a grading from 0 to 4 was assigned. Finally, the authors stated that, because KS lesions frequently showed significant intraspecimen heterogeneity, a score was calculated in which the percentage positive rating was multiplied by the intensity rating. Each component of the lesion was scored independently and the results were added up, so how were all these scorings were combined? Which are the scores indicated in Table 1-3? Finally, it seem that the scoring was done in the open and this may represent a bias.

3. In the Table summarizing the “Demographic, clinical and immunohistochemical scoring of HBD-3 and LL-37 of studied population” it would be important to indicate also the patient clinical stage.

4. Figure 1: arrows should be included in panels to point out positive cells, particularly in panels E and I. In addition, in order to correctly compare the expression of LL37 in plaque and nodules, panel I and J should have the same magnification.

5. The authors stated in Discussion that the expression of AMPs was increased with progression of KS lesions from plaque stage to nodules. However, to support of the possible role of these AMPs in progression of KS lesions from stage III and IV patients should be studied.

6. The manuscript does not cite a paper reporting that human beta defensin-2 (HBD-2) is expressed by endothelial cells present in Kaposi’s sarcoma lesions (but not by endothelial cells present in normal tissues), and that inflammatory mediators induce HBD-2 expression by normal endothelial cells Kawsar et al. (2010). This citation should be included in the manuscript and results discussed.

I have read this submission. I believe that I have an appropriate level of expertise to confirm that it is of an acceptable scientific standard, however I have significant reservations, as outlined above.

# Response

**Published:** 2012-12-17

**Authors:** Hanan Fathy

**Affiliations:** Faculty of Medicine, Mansoura University, Egypt

Firstly I thank Professor Barbara Ensoli for her valued comments. Below, I have some answers to the points she has made:

**1.** Patch lesions were not included in this study because it is very difficult to discriminate between tumor cells and activated endothelial cells in this stage of KS ( Reference number 29 in our manuscript).

**2.** In this study we used two different independent (i.e not added to each other) scores, one epidermal score and the other is dermal score as detailed in material and method section.

**3.**Tumor stage is presented in table 1. It is based on clinical examination and radiological study as mentioned in material and method section.

**4.** The panel i is replaced by another one with higher magnification (x400) and arrows are included.

**5.** Unfortunately none of the studied patients had stage III or IV.

**6.** We have included the findings of Kawsar et al. 2010 in the discussion.

Many Thanks.

# Peer review: Referee response for version 1

**Published:** 2012-11-02

**Authors:** Frank J Jenkins

**Affiliations:** 1 Department of Infectious Diseases and Microbiology, University of Pittsburgh, PA, USA

I have read this submission. I believe that I have an appropriate level of expertise to confirm that it is of an acceptable scientific standard.

# Peer review: Referee response for version 1

**Published:** 2012-11-02

**Authors:** Maxwell Fung

**Affiliations:** 1 Department of Dermatology, University of California, Davis, Sacramento, CA, USA

I have read this submission. I believe that I have an appropriate level of expertise to confirm that it is of an acceptable scientific standard.

# Peer review: Referee response for version 1

**Published:** 2012-10-31

**Authors:** Mohamed Badawy Abdel-Naser

**Affiliations:** 1 Klinik für Dermatologie, Venerologie und Allergologie Städtisches Klinikum Dessau, Dessau, Germany

Although I approve this paper, I have a few comments:

- The authors stated that “we performed a quantitative immunohistochemistry” however, it seems that they have done a qualitative assessment as quantitative immunohistochemistry is done by image analysis program which is reproducible, avoids interpersonal variations and potential bias. Furthermore, it provides quantitative data. If the authors wished to conduct quantitative immunohistochemistry then they would have to revise the statistical tests used.

- It seems that all authors evaluating the immunohistochemistry results were aware of the study design. This may be a potential source of bias.

- The authors used specimens of psoriasis as a positive control. Psoriasis is an inflammatory but not infective dermatosis which raises an argument against the impact of the expression of AMPs in KS.

I have read this submission. I believe that I have an appropriate level of expertise to confirm that it is of an acceptable scientific standard.

# Response

**Published:** 2012-11-12

**Authors:** Hanan Fathy

**Affiliations:** Faculty of Medicine, Mansoura University, Egypt

Firstly I thank Professor Naser for his valued comments. Below, I have some answers to the points he has made:

**1.** We performed a quantitative immunohistochemical analysis because the scoring of epidermal and dermal immunostain of the studied AMPs is a quantitative in nature and not a qualitative measure. We do agree that the image analysis program could be more accurate, but unfortunately this was not available to us. Furthermore, false positive results by positive staining of sweat glands (which is not a component of the tumor) with LL-37 can be obtained by the image analysis program used, so there is no need for reanalysis the data.

**2.** The specimens prepared hematoxylin, eosin and the immunohistochemical analysis were coded numerically. So the first two authors evaluating the immunohistochemistry results were unaware about the clinical data of the patients at the time of pathological evaluation. Even so, the stage of the disease (plaques versus nodules) can be determined by hematoxylin and eosin stained sections; Fernández-Figueras *et al.* ( Reference 29 in our manuscript).

**3.** Up until now the pathogenesis of KS has not been clear. HHV8 is considered to be a nec­essary, but not sufficient causal agent of KS. Apart from immuno­suppression and AIDS, additional cofactors still remain unknown ( Reference number 7 in our manuscript). This means that the etiology of KS is not just a viral infection. LL-37 was found to be highly expressed in malignant melanoma and squamous cell carcinoma (Kim *et al.* reference number 42 in our manuscript). Similarly, HBD-3 was found to be highly expressed in oral squamous cell carcinoma ( Kesting *et al*, Cancer Invest 2009, 27: 575-581). These AMPs have been found to be expressed in some inflammatory, infective and malignant diseases, so further research is needed to elucidate the biological function of these AMPs in KS.
